# Very Long-Term Follow-Up in Cardiac Resynchronization Therapy: Wider Paced QRS Equals Worse Prognosis

**DOI:** 10.3390/jpm11111176

**Published:** 2021-11-11

**Authors:** Patrick Leitz, Julia Köbe, Benjamin Rath, Florian Reinke, Gerrit Frommeyer, Christian Andresen, Fatih Güner, Julian Wolfes, Philipp S. Lange, Christian Ellermann, Lars Eckardt, Dirk G. Dechering

**Affiliations:** Department of Cardiology II—Electrophysiology, Albert-Schweitzer-Campus 1, University Hospital Muenster, 48149 Muenster, Germany; julia.koebe@ukmuenster.de (J.K.); benjamin.rath@ukmuenster.de (B.R.); florian.reinke@ukmuenster.de (F.R.); gerrit.frommeyer@ukmuenster.de (G.F.); christian.andresen@ukmuenster.de (C.A.); fatih.guener@ukmuenster.de (F.G.); julian.wolfes@ukmuenster.de (J.W.); philippsebastian.lange@ukmuenster.de (P.S.L.); christian.ellermann@ukmuenster.de (C.E.); lars.eckardt@ukmuenster.de (L.E.); dirk.dechering@niels-stensen-kliniken.de (D.G.D.)

**Keywords:** cardiac resynchronization, heart failure, very long-term follow-up, ECG, pacing

## Abstract

Background: Different electrocardiogram (ECG) findings are known to be independent predictors of clinical response to cardiac resynchronization therapy (CRT). It remains unknown how these findings influence very long-term prognosis. Methods and Results: A total of 102 consecutive patients (75 males, mean age 65 ± 10 years) referred to our center for CRT implantation had previously been included in this prospective observational study. The same patient group was now re-evaluated for death from all causes over a prolonged median follow-up of 10.3 years (interquartile range 9.4–12.5 years). During follow-up, 55 patients died, and 82% of the clinical non-responders (*n* = 23) and 44% of the responders (*n* = 79) were deceased. We screened for univariate associations and found QRS width during biventricular (BIV) pacing (*p* = 0.02), left ventricular (LV) pacing (*p* < 0.01), Δ LV paced–right ventricular (RV) paced (*p* = 0.03), age (*p* = 0.03), New York Heart Association (NYHA) class (*p* < 0.01), CHA_2_DS_2_-Vasc score (*p* < 0.01), glomerular filtration rate (*p* < 0.01), coronary artery disease (*p* < 0.01), non-ischemic cardiomyopathy (NICM) (*p* = 0.01), arterial hypertension (*p* < 0.01), NT-proBNP (*p* < 0.01), and clinical response to CRT (*p* < 0.01) to be significantly associated with mortality. In the multivariate analysis, NICM, the lower NYHA class, and smaller QRS width during BIV pacing were independent predictors of better outcomes. Conclusion: Our data show that QRS width duration during biventricular pacing, an ECG parameter easily obtainable during LV lead placement, is an independent predictor of mortality in a long-term follow-up. Our data add further evidence that NICM and lower NYHA class are independent predictors for better outcome after CRT implantation.

## 1. Introduction

Heart failure (HF) remains a leading cause of mortality in the western world [1]. Advances in pharmacological and device therapy have played a key role in improving survival and quality of life in patients with HF and reduced ejection fraction (HFrEF). Cardiac resynchronization therapy (CRT), which was first introduced over 30 years ago, has advanced to be a cornerstone of HFrEF therapy. A multitude of studies were able to demonstrate a positive effect of CRT therapy on mortality, quality of life, and left ventricular ejection fraction (LVEF) in patients with left bundle branch block (LBBB) [2,3]. However, there remains a significant portion of patients with a class I indication for CRT implantation, according to current guidelines, who do not benefit from CRT.

In a previous study [4] we were able to show electrocardiographic measurements such as shorter QRS duration during left ventricular (LV) pacing, and especially a shorter LV paced than right ventricular (RV) paced QRS width, to be independent predictors for short-term CRT response. Several consecutive studies have demonstrated a beneficial short-term effect of acute QRS narrowing in the presence of LBBB [5]. As long-term data on the effect of QRS changes during CRT pacing are sparse, we re-evaluated different electrocardiographic and clinical variables as predictors for very long-term survival in our previously described cohort [4] of HFrEF patients with an indication for CRT, according to the European Society of Cardiology (ESC) guidelines from that time.

## 2. Patients and Methods

### 2.1. Study Design and Data Collection

The present study is in accordance with regional and institutional ethics guidelines. The local ethics committee (Ethik-Kommission der Ärztekammer Westfalen-Lippe und der Westfälischen Wilhelms-Universität) approved the data collection. The primary endpoint was defined as death from any cause. Secondary endpoints were hospitalization due to heart failure. 

**Figure 1:**
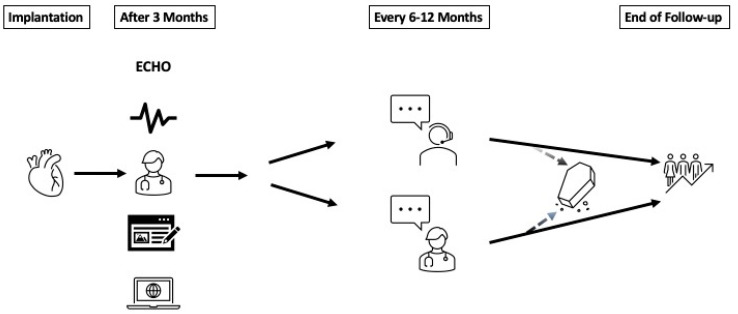
Follow-up design (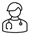: in person physician contact; 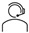: telephone physician contact; ECHO: trans-thoracic echocardiogram; 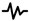: electrocardiogram; 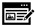: Minnesota Living with Heart Failure Questionnaire; 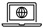: Device interrogation; 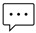: Interrogation on current NYHA class, hospitalizations; 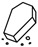: Deceased; 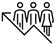: End of data collection and start statistical analysis).

Initially, all patients presented with persistent symptomatic heart failure (New York Heart Association (NYHA) class II–IV) despite optimal medical therapy and had a native QRS complex width >120 ms. Decision for CRT implantation was made according to the then applicable ESC guidelines [6]. The LV lead position was in all cases lateral or posterolateral. The follow-up design is represented in Figure 1.

### 2.2. Initial ECG Recordings

Standard supine 12-lead electrocardiogram (ECG) (50 mm/s, 10 mm/mV) results were obtained at baseline, perioperatively, and after 3 months. Intrinsic rhythm, LV paced, RV paced, and biventricular (BIV) paced ECG at a heart rate of 70 bpm, respectively, were documented. These recordings were then analyzed with regard to QRS duration using Datinf Measure software (Datinf GmbH, Tübingen, Germany). Regarding QRS width, only the widest QRS complex of the 12-lead ECG was used for analysis. 

### 2.3. Definition of CRT Responders

Response to CRT was defined as improvement in NYHA class > 1 based upon the Minnesota Living with Heart Failure Questionnaire. Non-responders were defined as lack of improvement or even worsening of NYHA class and/or at least one heart failure hospitalization with intravenous diuretic use. In order to reassess adequate resynchronization, all patients classified as non-responders were re-evaluated by echocardiography at 3- or 6-months follow-up. Optimization of device programming was done according to a stepwise approach [7].

### 2.4. Definition of Ischemic Cardiomyopathy vs. Non-Ischemic Cardiomyopathy

Ischemic cardiomyopathy (ICM) was defined as a reduction in LVEF due to a history of MI or revascularization (coronary artery bypass grafting (CABG) or percutaneous coronary intervention (PCI)), or patients with ≥75% stenosis of the left main or proximal left anterior descending coronary artery (LAD), or patients with ≥75% stenosis of two or more epicardial vessels. Patients with single-vessel disease and no prior history of revascularization or myocardial infarction (MI) were classified as non-ischemic (NICM).

### 2.5. Long-Term Follow-Up Collection

Long-term follow-up data were gathered from our outpatient clinic, where patients were seen every 6–12 months. Patients who were not regularly seen in our clinic were contacted by phone. In these cases, their treating physician was also contacted. During the visits/telephone interviews, patients’ data on the current NYHA class and hospitalizations, including the reason for hospitalization, were retrieved. If a patient missed a follow-up visit and could not be reached by phone, the general physician was contacted. All patients’ deaths were confirmed by the treating general physician. Data were collected from the electronic hospital information system.

### 2.6. Statistical Analysis

Statistical analysis was performed using SPSS (Version 25, IBM SPSS Statistics, IBM Corporation, Armonk, NY, USA). Continuous data were expressed as mean ± SD and compared between groups using a Mann–Whitney U Test. Categorical data were summarized by their observed frequencies and percentages, and compared using cross tabulation and a Chi-square Test. 

Univariable and multivariable Cox regression analyses were performed to determine the parameters associated with very long-term survival. Associations with survival were further described with hazard ratios and 95% confidence intervals. The multivariable Cox regression model included variables with univariate *p* < 0.05. Additionally, clinical characteristics with known association with survival in the described cohort were forced into different multivariable models, regardless of the univariate *p* value. To avoid intercorrelations, only one of the ECG parameters was forced into each model. Only parameters that showed a significant association throughout all of the constructed models were labelled as significant predictors. Finally, survival rates were depicted with Kaplan–Meier curves with their corresponding tables showing the number of patients at risk at different corresponding points of follow-up. For all statistical tests a value of *p* < 0.05 was considered significant.

## 3. Results

### 3.1. Baseline Characteristics

The basis for the presented analysis is the previously described cohort [4]: A total of 102 consecutive patients referred to our center for CRT implantation were initially enrolled, and 14 patients were lost to follow-up at some point. Two patients presented with a right bundle branch block (RBBB) masking LBBB. Native QRS duration at implantation was 166 ± 33 ms. The mean age of the cohort was 70 ± 10 years old at the time of implantation; 66% of the patients were male. Further baseline characteristics of our population, as well as the ECG measurements, are listed in Table 1.

### 3.2. Follow-Up Duration

The first CRT implantation enrolled in the study took place on 1 January 2004, and follow-up ended for all patients on 22 September 2019. The shortest censored time was 8 years, 6 months, and 7 days. The longest censored time was 15 years, 2 months, and 26 days. Median follow-up was 10.3 years (interquartile range 9.4–12.5 years).

### 3.3. Adverse Events

During a total of 924 years of patient follow-up, three patients developed device-related infections with the need of antibiotic therapy. Two patients suffered a stroke, one patient underwent transcatheter aortic valve replacement, and one patient had a ST elevation myocardial infarction (STEMI). Due to terminal heart failure, three patients underwent left ventricular assist device implantation to bridge for heart transplantation. All three patients received a heart transplant further down the line.

### 3.4. Survival

Survival censored at 36, 60, 96, and 120 months was 89%, 83%, 62%, and 51%, respectively. Median survival was 126 months.

### 3.5. Possible Predictors of Long-Term Outcomes

Results from the univariate and multivariate Cox regression for the primary and secondary endpoints are found in Table 2. 

Regarding our primary endpoint, using a univariate analysis of age (*p* = 0.03), NYHA class (*p* < 0.001), CHA_2_DS_2_-Vasc score (*p* < 0.001), glomerular filtration rate (GFR) (*p* = 0.007), arterial hypertension (*p* = 0.004), presence of coronary artery disease (*p* = 0.001), presence of non-ischemic (versus ischemic) cardiomyopathy (NICM; *p* = 0.01), clinical response to resynchronization therapy (*p* = 0.008), and NT-proBNP (*p* < 0.001), the results showed a significant association with survival. 

Of the electrocardiographic parameters, native QRS width and RV paced values failed to reach statistical significance in the univariate analysis.

Multivariate analysis retained NYHA class (*p* = 0.03), presence of NICM (*p* = 0.003), and QRS width during BIV stimulation (*p* = 0.01) as independent predictors of survival. 

**Figure 2:**
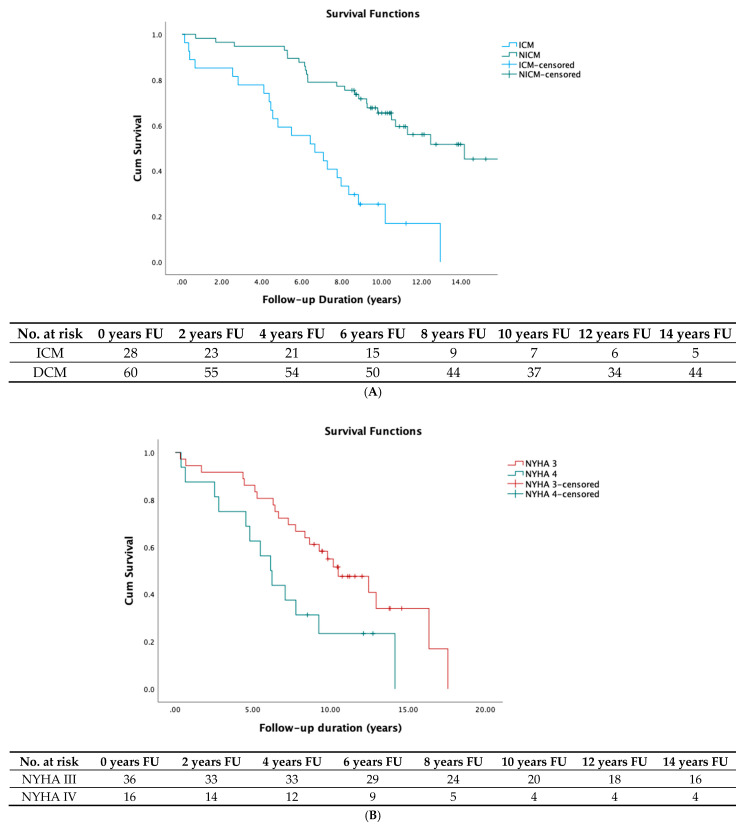

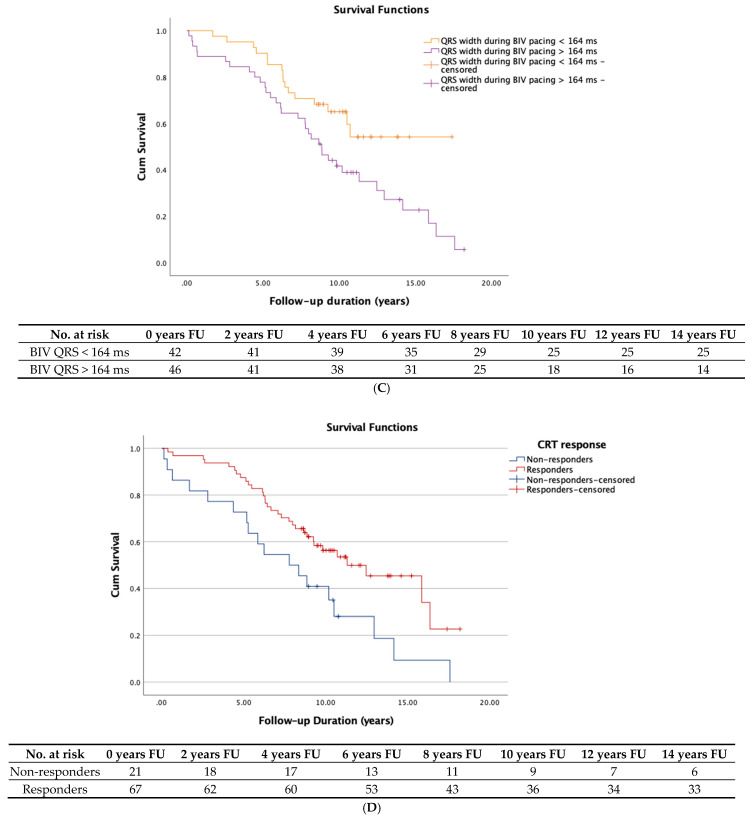
Kaplan–Meier survival functions, with corresponding tables describing the number (N) of patients at risk at a given point of the follow-up, for presence of NICM vs. ICM (Panel **A**); patients with NYHA III vs. NYHA IV at the time of implantation (Panel **B**). Patients with upper vs. lower median of QRS width during biventricular pacing (Panel **C**), as well as clinical responders to CRT therapy vs. non-responders to CRT therapy (Panel **D**). (NICM: non-ischemic cardiomyopathy; NYHA: New York Heart Association; BIV: biventricular pacing; ICM: ischemic cardiomyopathy; Cum survival: cumulative survival; ms: milliseconds; CRT: cardiac resynchronization therapy).

We further performed stratified analysis to evaluate the robustness of our data. QRS width during BIV stimulation in the subgroups of men (*p* = 0.04), patients with wider native QRS (*p* = 0.006) throughout all age groups (*p* = 0.04 in the younger (<74 years) and *p* = 0.05 for the elder half group) remained statistically significant. NYHA class showed significant associations only in men (*p* = 0.01) and the younger half group (*p* < 0.04). Finally, NICM remained statistically significant only in the elderly (*p* = 0.02). Kaplan–Meier curves for survival are shown in Figure 2. Concerning the secondary endpoint in the multivariate Cox regression analysis, only the NYHA class at the time of implantation revealed to be an independent predictor of hospitalization. None of our ECG parameters reached statistical significance in the multivariate Cox model.

## 4. Discussion

In the present study, a shorter QRS width during biventricular pacing in the first months after CRT implantation, as well as lower NYHA class and the presence of non-ischemic (compared to ischemic) cardiomyopathy, independently predicted cardiovascular survival in patients with class I indication for cardiac resynchronization therapy over a very long-term follow-up. Other ECG parameters that were shown to predict short-term clinical response to CRT (i.e., QRS width of LV or RV pacing, native QRS width, difference between LV and RV paced QRS) did not predict long-term survival. Of note, shorter QRS width during biventricular pacing did not predict a short-term clinical response in our original paper.

Several studies were able to demonstrate the beneficial effect of QRS narrowing on CRT response rate, survival, and ventricular remodeling [8,9]. A recent meta-analysis of 32 studies from Bazoukis et al. sought to review the association between QRS narrowing through CRT and clinical, as well as echocardiographic, response [10]. The quantitative synthesis showed that patients with clinical improvement exhibited shorter QRS durations through CRT. The same was seen with echocardiographic response, although, in contrast to clinical response, not all of the retained studies showed favorable results.

Our cohort adds further positive data on the effect of QRS narrowing through CRT on very long-term survival. In daily clinical practice, this simple ECG parameter might be of particular value, due to being readily available, in contrast to more sophisticated and costly diagnostics [11], with little interobserver variability. Our data may thus be of clinical relevance as they are easily integrable in the implantation routine in cases where more than one target vein can be selected.

The quest for optimal QRS narrowing should not stop with lead placement. Recent data of Verma et al. showed the importance of a patient-tailored individual device programming to obtain optimal QRS narrowing [12]. In a well-selected collective, with optimal LV lead placement, using a device-based algorithm, which automatically adjusted the paced atrioventricular delay, the authors obtained QRS narrowing during BIV pacing, regardless of underlying cardiomyopathy or the native QRS width. Further, as proposed by Jastrzebski et al., in patients where QRS narrowing through CRT cannot be achieved, despite optimal programming, HIS bundle or left bundle branch pacing seems to be an attractive option [13]. Although long-term data on hard endpoints are lacking, QRS narrowing might not be beneficial for all patients. Previous data showed only patients with LBBB seemed to benefit from device-induced QRS narrowing [13].

Our multivariate analysis also revealed the presence of NICM to be an independent predictor of survival over our very long follow-up. Moreover, we were able to show the independent predictive value of the patients’ NYHA class for long-term survival. In short, up to median duration follow-up, several previous studies also reported patients´ NYHA classes and their underlying cardiomyopathy to be independent predictors of survival in CRT [14,15,16,17]. The superior response to CRT in NICM and in patients with a lower burden of symptoms has been known since the first randomized CRT trials, like MADIT-CRT, albeit over a shorter follow-up period. The landmark MADIT-CRT trial showed cardiac resynchronization therapy with defibrillator (CRT-D) to reduce rates of mortality or heart failure events when compared to implantable cardioverter defibrillator (ICD) placement alone among heart failure patients with LVEF ≤ 30% and QRS duration ≥130 msec [18]. This benefit is primarily driven by a reduction in HF events. The cohort of 1820 patients comprised patients with a lower NYHA class (NYHA I-II exclusively) and a greater percentage of ICM, in comparison to our population. A secondary analysis of MADIT-CRT found patients with NICM and lower NYHA class to show a greater response to CRT [19]. The 7-year follow-up results from the trial revealed a cumulative rate of all-cause mortality for the CRT patients of 18% [20]. At the 7-year follow-up, we saw a cumulative rate of all-cause mortality of 31%, which can be attributed to the advanced age and higher NYHA class of our cohort. Nearly half of our elderly patient population were still alive at the end of our median follow-up of 10.3 years. As novel drug developments (e.g., the landmark DAPA-HF and PARADIGM-HF trials) boost survival through new pharmacological therapies, long-term survival rates will likely increase in the future [21,22].

Of note, although response to CRT and NT-proBNP showed in the univariate analysis a strong correlation with outcome, it did not retain its statistical significance in our multivariate model. We opted to define response to CRT using clinical improvement in NYHA class; however, in the published literature there is a lack of consensus on the definition of response to CRT [23]. The predictive value of the different criteria used to define response to CRT varies considerably. Boidol et al. were able to show clinical characteristics to exhibit a higher sensitivity, whereas echocardiographic measurements showed a higher specificity in the context of predicting survival [24]. Their data from the TRUST CRT trial found improvement in NYHA class to be a powerful predictor of outcome. However, due to multiple interactions with baseline characteristics, the accuracy varied significantly throughout different subgroups.

Finally, device-related complications remain a major cause of morbidity in CRT patients. In our cohort, we saw comparably low complication rate of 2.9% over the complete follow-up (three patients with device-related endocarditis). Previous data from the Italian Clinical Service project reported rates of device-related infections of 0.9 events per 100 patient-years after the first implantation, and 1.8 events per 100 patient-years after the device replacement procedure [25]. None of our subjects presented recurring infections after device replacement.

### Limitations

A major limitation to our study is due to its design. We present a prospective observational study without randomization with a relatively small sample size. Due to the non-continuous follow-up, we do not have sufficient data on the time varying and the disease or on the survival modifying factors and we cannot account for an eventual interference with our potential predictors for survival. However, we prospectively evaluated a real-world CRT population over a very long period of time.

## 5. Conclusions

During very long-term follow-up, we were able to add further evidence relative to the importance of QRS shortening through CRT. The easily obtainable ECG parameters should be one of the main endpoints of resynchronization. LV lead placement, as well as device programming, should be adjusted to obtain maximal QRS shortening. Our data strengthen the status of the underlying cardiomyopathy, as well as the NYHA class, in patient selection for resynchronization therapy.

## Figures and Tables

**Table 1:** Patient characteristics at the time of implantation and ECG parameters (NYHA: New York Heart Association; GFR: glomerular filtration rate; NICM: non-ischemic cardiomyopathy; ng/L: nanogram per liter; LVEF: left ventricular ejection fraction; BIV: bBiventricular; LV: left ventricular; RV: right ventricular; Δ: difference; ms: milliseconds; kg: kilogram).

	Survivors *n* = 36	Deceased *n* = 52	Entire Population *n* = 102	*p*-Value
Age (Years)	70 ± 10	76 ± 9	74 ± 10	0.03
Weight (kg)	86 ± 16	86 ± 19	85 ± 19	0.56
NYHA Class	2.7 ± 1	3.17 ± 1	3.4 ± 1.2	<0.001
CHA_2_DS_2_-Vasc	3.4 ± 2.7	5.5 ± 2.5	4.7 ± 2.7	<0.001
GFR (mL/min)	56.7 ± 6.7	48.3 ± 12.8	51.4 ± 11.6	0.007
Atrial Fibrillation (%)	47.4	49.1	47	0.94
Arterial Hypertension (%)	50	61.8	62	0.116
Type II Diabetes (%)	26.3	29.1	28	0.818
Coronary artery disease (%)	23.7	60	45	0.01
NICM (%)	86.5	51.9	67.3	0.01
Male Gender (%)	65.8	85.5	75	0.042
Clinical Responder (%)	89.5	67.3	77.5	0.014
LVEF (%)	30.7 ± 12.3	28.8 ±7.7	29.4 ± 9.7	0.77
NT-proBNP (ng/L)	1534 ± 1781	5339 ± 7676	3658 ± 6086	0.02
No pacing (ms)	162.6 ± 33	170.3 ± 34.8	166.6 ± 33.3	0.173
BIV pacing (ms)	149.3 ± 27.8	173.7 ± 30.2	162.0 ± 31.4	<0.001
LV pacing (ms)	177.5 ± 33.8	202 ± 40.4	191.5 ± 38.4	<0.001
RV pacing (ms)	199.9 ± 28.3	210.7 ± 34.7	205.7 ± 32.4	0.035
Δ LV paced–RV paced (ms)	−22.4 ± 37.2	−8.7 ± 30.8	−14.5 ± 33.6	0.078

**Table 2:** Uni- and multivariate Cox regression for the primary and secondary endpoints of patient characteristics at the time of implantation and ECG parameters (BIV: bBiventricular; LV: left ventricular; RV: right ventricular; Δ: difference; NYHA: New York Heart Association; GFR: glomerular filtration rate; NICM: non-ischemic cardiomyopathy; ng/L: nanogram per liter; LVEF: left ventricular ejection fraction; HR: hazard ratio; 95% CI: 95% confidence interval).

	Primary Endpoint	Primary Endpoint	Secondary Endpoint	Secondary Endpoint
	*p*-Value Univariate Cox Regression {HR (95% CI)}	*p*-Value Multivariate Cox Regression {HR (95% CI)}	*p*-Value Univariate Cox Regression {HR (95% CI)}	*p*-Value Multivariate Cox Regression {HR (95% CI)}
QRS width with no pacing	0.09 {1.01(0.99–1.02)}		0.3 {1.00(1.00–1.01)}	
QRS width during BIV pacing	0.02 {2.51(1.39–4.57)}	0.01 {3.89(1.36–11.14)}	0.05 {1.57(1.00–2.49)}	
QRS width during LV pacing	<0.001 {1.01(1.01–1.02)}		0.006 {1.01(1.00–1.02)}	
QRS width during RV pacing	0.09 {1.01(1.00–1.02)}		0.12 {1.01(0.99–1.01)}	
ΔQRS width during LV paced– QRS width during RV paced	0.03 {1.01(1.00–1.02)}		0.19 {1.00(0.99–1.01)}	
Age (Years)	0.03 {1.04 (1.00–1.07}		0.07 {1.02(0.99–1.04)}	
Male Gender (%)	0.074 {0.48(0.21–1.07)}		0.1 {0.63(0.36–1.09)}	
Weight (kg)	0.52 {0.99 (0.98–1.01)}		0.56 {1.00(0.99–1.02)}	
NYHA Class	<0.001 {2.40 (1.40–4.13)}	0.03 {2.46(1.08–5.58)}	0.01 {1.68(1.31–2.48)}	0.04 {1.71(1.02–2.88)}
CHA_2_DS_2_-Vasc	<0.001 {1.24(1.10–1.40)}		0.07 {1.13(1.03–1.23)}	
GFR (ml/min)	0.007 {0.96(0.93–0.98)}		0.19 {0.98(0.96–1.01)}	
Atrial Fibrillation (%)	0.94 {0.75(−0.53–0.69)}		0.97{1.01(0.64–1.59)}	
Arterial Hypertension (%)	0.004 {1.62(1.17–2.25)}		0.11 {1.31(0.94–1.81)}	
Type II Diabetes (%)	0.818 {0.94(−0.70–0.63)}		0.922 {1.03(0.62–1.70)}	
Coronary artery disease (%)	0.001 {2.81(1.56–5.05)}		0.002 {2.04(1.29–3.23)}	
NICM (%)	0.01 {0.31(0.17–0.56)}	0.003 {0.23(0.09–0.60)}	0.004 {0.49(0.30–0.80)}	
Clinical Responder (%)	0.008 {0.44(0.24–0.81)}	*0.77 {0.85(0.27–2.63)}*	0.03 {0.56(0.32–0.95)}	
LVEF (%)	0.56 {0.99(0.95–1.03)}		0.30 {0.99(0.96–1.01)}	
NT-proBNP (ng/L)	<0.001 {1.0(1.0–1.0)}		<0.001 {1.0(1.0–1.0)}	

## Data Availability

The datasets used and/or analysed during the current study are available from the corresponding author on reasonable request.
